# Refuges and ecological traps: Extreme drought threatens persistence of an endangered fish in intermittent streams

**DOI:** 10.1111/gcb.15116

**Published:** 2020-05-18

**Authors:** Ross Vander Vorste, Mariska Obedzinski, Sarah Nossaman Pierce, Stephanie M. Carlson, Theodore E. Grantham

**Affiliations:** ^1^ Department of Environmental Science, Policy, & Management University of California Berkeley Berkeley CA USA; ^2^ California Sea Grant Windsor CA USA; ^3^ Present address: Department of Biology University of Wisconsin La Crosse La Crosse WI USA

**Keywords:** abiotic, isolated pools, mixed models, mortality, Pacific salmon, river drying, threatened species, water abstraction

## Abstract

Recent droughts raise global concern over potential biodiversity loss and mitigating impacts to vulnerable species has become a management priority. However, drought impacts on populations are difficult to predict, in part, because habitat refuges can buffer organisms from harsh environmental conditions. In a global change context, more extreme droughts may turn previously suitable habitats into ecological traps, where vulnerable species can no longer persist. Here, we explore the impacts of California's recent record‐breaking drought on endangered juvenile Coho salmon. We estimated the variability of cumulative salmon survival using mark–recapture of nearly 20,000 tagged fish in intermittent stream pools during a 7‐year period encompassing drought and non‐drought conditions. We then determined the relative importance of physical habitat, streamflow, precipitation, landscape, and biological characteristics that may limit survival during drought. Our most striking result was an increase in the number of pools with reduced or zero survival during drought years and a coincident increase in spatial variability in survival among study reaches. In nearly half of the stream pools, salmon survival during drought was similar to mean survival of pools assessed during non‐drought years, indicating some pools had remarkable resistance (ability to withstand disturbance) to extreme drought. Lower survival was most attributable to longer duration of disconnection between upstream and downstream habitats, a consequence of increasing drought severity. Our results not only suggest that many pools sustain juvenile salmon in non‐drought years transition into ecological traps during drought but also highlight that some pools serve as refuges even under extreme drought conditions. Projected increases in drought severity that lead to longer droughts and greater habitat fragmentation could transform an increasing proportion of suitable habitats into ecological traps. Predicting future impacts of drought on Coho salmon and other sensitive species will require identification and protection of drought refuges and management strategies that prevent further habitat fragmentation.

## Graphical abstract

We explored the impacts of California's recent record‐breaking drought on endangered juvenile Coho salmon by estimating survival of nearly 20,000 tagged fish in intermittent stream pools during a 7‐year period. As drought conditions became extreme, there was an increase in the number of pools with reduced or zero survival and variability of survival estimates. This suggests that many pools that sustain juvenile salmon in non‐drought years transition into ecological traps during drought, but also highlights that some intermittent streams serve as refuges even during extreme drought.

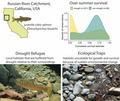

## 1 INTRODUCTION

Droughts are an increasing threat to ecosystems worldwide, with unprecedented multi‐year droughts recently observed in California, Australia, and South Africa (Robeson, 2015; van Dijk et al., 2013; Vogel & van Zyl, 2016). Harsh environmental conditions associated with droughts can lead to dramatic changes in the composition and abundance of biota, as well as species extirpations (e.g., Bogan & Lytle, 2011; Matusick, Ruthrof, & Hardy, 2012). However, the impact of droughts on ecosystems can vary greatly across the landscape (Nimmo, Haslem, Radford, Hall, & Bennett, 2016). Habitat refuges, which are defined as areas buffered from disturbance relative to their surroundings, can reduce the short‐term impacts of drought on biota (Davis, Pavlova, Thompson, & Sunnucks, 2013; Keppel et al., 2012). Therefore, accurate predictions of drought‐induced changes in biota will ultimately depend on identifying drought refuges and assessing their ability to support species survival under more extreme drought conditions.

Human activities can induce ecological traps, or habitats that were once preferentially used by biota that no longer support growth and survival due to sudden environmental changes (Robertson & Hutto, 2006; Schlaepfer, Runge, & Sherman, 2002). For example, juvenile African penguins follow environmental cues, such as water temperature, to productive feeding habitats; however, climate change and industrial fishing have depleted productivity in these habitats such that temperature cues are no longer correlated with food supplies (Sherley et al., 2017). This ecological trap has contributed to an ~80% reduction in the affected penguin population (Sherley et al., 2017). Biological communities in river ecosystems, such as fish, are also susceptible to ecological traps, especially when river flow regimes are altered by anthropogenic activities. For example, Coho salmon (*Oncorhynchus kisutch*) in the Shasta River of northern California successfully spawn in river reaches that now become inhospitable during the juvenile rearing period as a consequence of increased human water withdrawals. These reaches have been transformed from suitable dry‐season rearing habitat into ecological traps for this highly endangered fish population (Jeffres & Moyle, 2012). Yet, there remains relatively little empirical evidence on which environmental factors cause suitable habitat to become ecological traps for biota or of the population‐level implications of such transformations (Hale & Swearer, 2016).

Residual pools in intermittent streams are a habitat that may be vulnerable to become ecological traps due to human activities, including water withdrawals and habitat modification. Intermittent streams, defined by their predictable cessation of flows during dry periods, are globally ubiquitous, and support a high diversity of freshwater and terrestrial species (Datry et al., 2018), including at‐risk species (Labbe & Fausch, 2000; Wall, Berry, Blausey, Jenks, & Kopplin, 2004; Wigington et al., 2006). For example, intermittent and ephemeral streams comprise up to 66% of the river network in California (Levick et al., 2008) and are inhabited by endangered juvenile Coho salmon, a species of cultural and commercial importance (Lichatowich, 1999). Despite the harsh environmental conditions, Coho salmon growth and survival have been found to be higher in intermittent than perennial streams, presumably due to lower fish density and higher food resources (Wigington et al., 2006). During typical summer low‐flow conditions, residual pools are important for juvenile salmon as they are the only suitable aquatic habitat remaining within intermittent stream reaches (Hwan & Carlson, 2016). However, extreme drought and human water withdrawals can have the potential to exacerbate stream drying, creating harsher environmental conditions and causing pools to dry. Salmon trapped in drying streams can experience reduced survival, likely due to declines in dissolved oxygen as well as increased water temperatures, competition, and/or predation (Grantham, Newburn, Mccarthy, & Merenlender, 2012; Hwan, Fernández‐Chacón, Buoro, & Carlson, 2018; Obedzinski, Nossaman, Horton, & Deitch, 2018; Woelfle‐Erskine, Larsen, & Carlson, 2017). Both broad‐scale climatic factors, such as drought severity and local‐scale anthropogenic activities, appear to control the balance between intermittent stream habitat serving as drought refuges or acting as ecological traps for salmon (Jeffres & Moyle, 2012; Magoulick & Kobza, 2003).

In this study, we addressed the question of how extreme drought influences species survival in intermittent streams and identified the primary environmental factors controlling the occurrence of refuges and ecological traps. We used mark–recapture techniques to estimate over‐summer survival of juvenile Coho salmon in intermittent streams during one of northern California's most severe droughts on record (Robeson, 2015). We measured pool‐scale (i.e., habitat‐unit scale) survival within four to eight stream reaches across four tributaries over 7 years (2011–2017). We predicted that salmon survival during extreme drought would be reduced compared to survival during non‐drought years. We also predicted that variability in survival across the study region would increase during drought due to the transformation of some pools into ecological traps (i.e., habitats with reduced survival during drought years) during extreme drought. We then determined the relative importance of environmental variables on survival, including precipitation, landscape variables, water quality, streamflow, and physical habitat variables using a mixed modeling framework. We predicted that streamflow and habitat fragmentation would be the most influential variables in explaining variation in salmon survival.

## 2 MATERIALS AND METHODS

### 2.1 Study reaches

**FIGURE 1:**
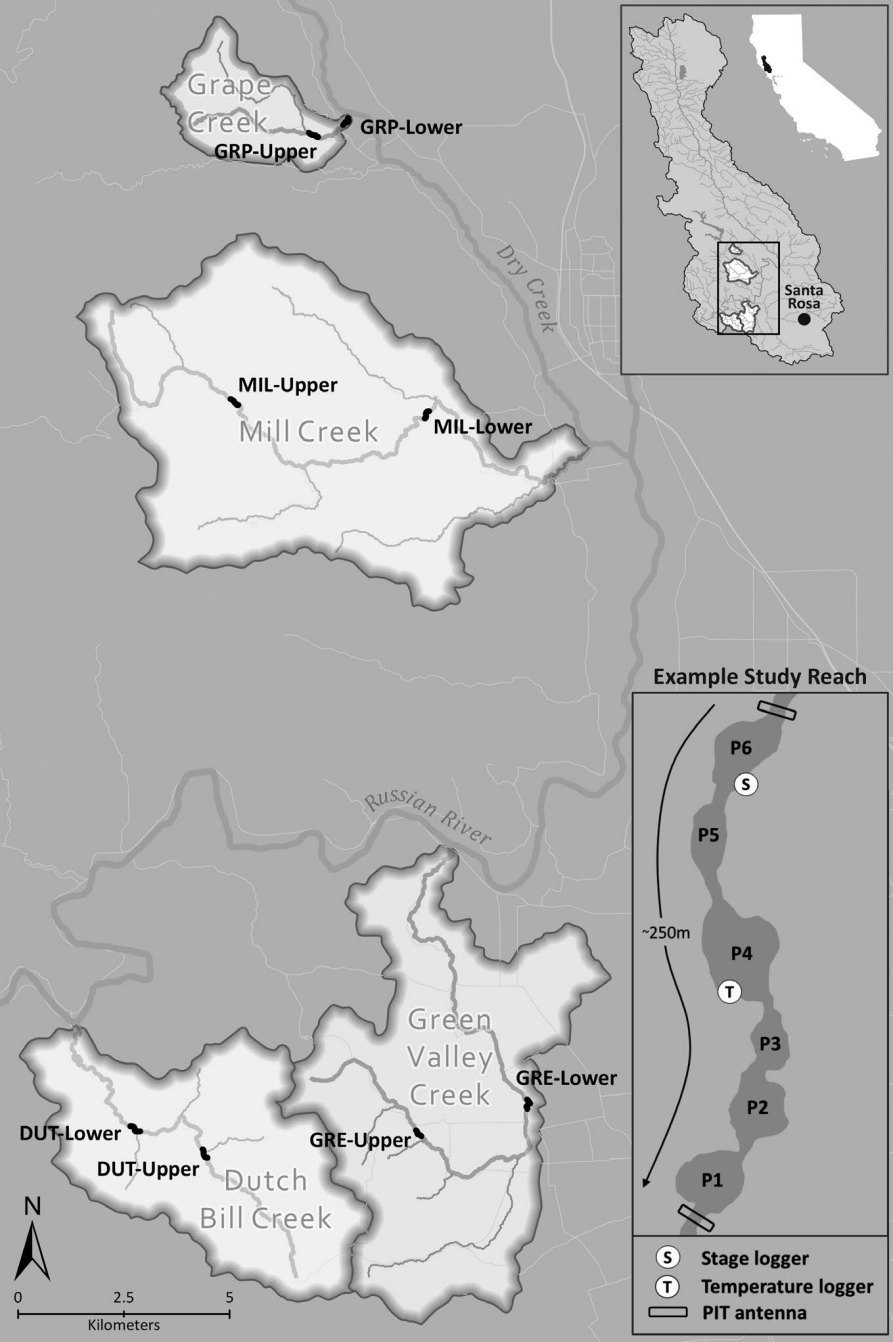
Map of four tributaries and eight study reaches within the Russian River catchment (upper right inset), including a diagram (lower right inset) of an example study reach. Study reach example shows location of pool habitats (P), temperature (T) and stage (i.e., streamflow) loggers (S), and PIT‐tag antennas

Between 2011 and 2017, we surveyed reaches of Dutch Bill (DUT), Green Valley (GRE), Mill (MIL), and Grape (GRP) creeks, tributaries of the Russian River in northern California (Figure 1; Table 1). The Russian River catchment is characterized by a Mediterranean climate (23°C [21–24°C] 30‐year mean [range] daily maximum air temperature, 990 mm [169–1,740 mm] 30‐year mean [range] of annual precipitation total, Healdsburg, CA) in which nearly all precipitation occurs in the form of rainfall between November and April. This results in peak streamflow during the winter season that slowly recedes through the spring and summer, leading many small tributaries to cease to flow or dry completely. Vineyards and rural residential homes occupy much of the landscape, and streamflow is influenced by water withdrawals from small‐scale direct diversions and streamside wells (Deitch, Kondolf, & Merenlender, 2009). Common fish species occurring within the study reaches included Coho salmon, steelhead trout (*O. mykiss*), sculpin (*Cottus* spp.), and roach (*Hesperoleucus* spp.).

**TABLE 1:** Site characteristics for eight study reaches within the Russian River catchment

Study reach	Length (m)	Catchment area (km^2^)	Slope (%)	Geomorphic type	Cropland area (%)	Mean (range) pool disconnection (days)^a^
DUT lower	290	24.8	0.4	Alluvial	4	13 (0–37)
DUT upper	260	14.9	1.3	Bedrock	7	4 (0–14)
GRE lower	310	43	0.5	Alluvial	20	5 (3–7)
GRE upper	220	8	0.3	Clay	2	29 (0–78)
GRP lower	230	8.2	0.7	Alluvial	13	14 (0–71)
GRP upper	230	7.4	1.9	Bedrock	11	41 (27–69)
MIL lower	210	30	0.7	Bedrock	1	7 (0–18)
MIL upper	240	10.4	1.1	Bedrock	3	2 (0–11)

Abbreviations: DUT, Dutch Bill; GRE, Green Valley; GRP, Grape; MIL, Mill.

^a^ Approximate number of days with mean streamflow below 0.28 L/S (0.01 ft^3^/s) from June 1 to October 31 each year from 2011 to 2017.

We selected two reaches within each of the four study streams based on Coho salmon habitat suitability, varying levels of flow impairment, and permission to access the streams from private landowners. Initial habitat surveys were conducted to define reach boundaries and classify habitat units (pools, riffles, flatwaters) with the intention of selecting suitable habitat for salmon survival. Each reach was approximately 250 m long (mean [range] = 249 m [210–310 m]) and contained 3–12 pools (mean = 7.4 pools).

### 2.2 Drought classification

The period 2012–2016 was an historic drought that affected most of California, including the study region. We described drought conditions in the Russian River catchment using data from the US Drought Monitor (https://droughtmonitor.unl.edu/), which classifies drought based on key indicators including streamflow, soil moisture, and precipitation.

### 2.3 Study population

The Russian River catchment once supported a large, self‐sustaining population of Coho salmon; however, due to habitat loss and degradation, the population was nearly extirpated by the late 1990s. Russian River Coho salmon are part of the Central California Coast Evolutionary Significant Unit, which was listed as federally endangered in 2005 (70 FR 37160). Recovery strategies include propagation of juvenile Coho salmon in a local conservation hatchery. Releases of hatchery‐reared juvenile Coho salmon into study streams provided an opportunity to compare over‐summer survival in relation to environmental variables using a common population of experimental fish similar in size and genetic composition.

### 2.4 Biological data collection

Each year, approximately 500 hatchery‐reared juvenile Coho salmon (*n* = 19,666 total) implanted with passive integrated transponder (PIT) tags, were released into study reaches in mid‐June and tracked using stationary and portable PIT‐tag detection systems through early October. Stationary antennas were placed at reach boundaries to document fish moving outside of the study reach and portable antennas allowed us to detect movement within stream pools inside the study reaches. Depending on available resources, between four and eight reaches were stocked and surveyed each year. Fish were not stocked into reaches when observed spring habitat conditions indicated that they would have no chance of survival, such as when pools were already disconnected and drying in June. We revisited these unstocked reaches at the end of summer to confirm the absence of wetted habitat and validate the assumption that fish survival would have been zero. To estimate the habitat‐unit‐specific survival over multiple intervals between June and October, we conducted a series of surveys using a portable PIT‐tag detection method (O'Donnell, Horton, & Letcher, 2010). A total of three to five surveys were done approximately monthly during each summer, depending on reach and year. Each survey consisted of two site visits on consecutive days allowing estimation of abundance during each survey and survival between surveys while accounting for capture probability following the robust design mark–recapture model (Kendall, Nichols, & Hines, 1997; Obedzinski et al., 2018).

### 2.5 Environmental data collection

Between June and October (2011–2017), we collected data on physical habitat, streamflow, water quality, precipitation, and landscape characteristics as potential explanatory variables for predicting over‐summer survival. Within each habitat unit (i.e., stream pool), we measured physical habitat dimensions (depth, length, width, and volume), water temperature, and dissolved oxygen levels during monthly sampling occasions. Dissolved oxygen and temperature were measured using a handheld device consistently between 08:50 and 11:30 hr to minimize diel variation. We also measured water temperature continuously (60 min intervals) in one habitat‐unit per reach, chosen to represent the physical and hydrologic characteristics of all units within the reach. Streamflow was measured approximately once per month to generate reach‐specific rating curves based on correlation with continuous stage readings at loggers within each reach, following Rantz (1982). From these streamflow data, we calculated summary statistics related to minimum, maximum and mean daily flow, and number of days of disconnection among habitat units, estimated to occur in these reaches when streamflow falls below 0.28 L/S (0.01 ft^3^/s; Obedzinski et al., 2018). Percent of cropland area within the contributing catchment of each reach was calculated using GIS software (ArcPro 2.2; Esri) based on data provided by Sonoma County Vegetation Mapping and LiDAR Program (http://sonomavegmap.org). We calculated precipitation sums for the duration of the survival intervals and antecedent precipitation in the water year prior to stocking events (October–May) using monthly catchment averages of precipitation obtained from the PRISM database (PRISM Climate Group, Oregon State University, http://prism.oregonstate.edu).

### 2.6 Data analysis

#### 2.6.1 Habitat‐unit‐level fish survival estimates using mark–recapture

To estimate the survival between each sampling occasion in each habitat unit, we first used PIT‐tag detections from paired wand surveys to construct an encounter history for each individual, and then applied the robust design mark–recapture model (Kendall et al., 1997) in program MARK (White & Burnham, 1999). Program MARK uses general linear modeling to estimate beta parameters, which are combined using a sub‐model (e.g., sin link, logit link) to estimate real parameters of interest (i.e., survival). If a fish moved from one habitat unit to another over the course of the summer study period, it was included in the interval‐specific survival estimates for its original habitat unit the date it was detected in a new habitat unit, after which it was included in the survival estimates for the new habitat unit. If a fish was detected leaving the study reach all together, it was excluded from survival estimates for all intervals following the date that it was detected leaving (*n* = 1,476 fish or 7.5% of all fish). Survival estimates generated for each habitat unit were multiplied across all sampling intervals within a season to estimate cumulative over‐summer survival each year.

#### 2.6.2 Assessing distribution of survival estimates during drought and non‐drought years

We assessed the distribution of survival estimates from drought (2012–2016) and non‐drought (2011 and 2017) years using probability density functions to account for non‐normal distributions and to avoid binning data. We tested the equality of distributions of the two groups using the *sm.density.compare* function in the *sm* package in R (Bowman & Azzalini, 2014).

#### 2.6.3 Characterizing stream pools as refuges and ecological traps for juvenile salmon

To characterize refuges and ecological traps, we first quantified the mean cumulative survival observed across all study reaches in non‐drought years. We then evaluated pool‐scale survival in drought years relative to this threshold. Refuges were defined as pools that during drought had survival greater than or equal to one standard deviation of the mean survival threshold from non‐drought years. Ecological traps were defined as pools that during drought had survival less than one standard deviation of the threshold value.

#### 2.6.4 Determining effects of explanatory variables on survival estimates

We selected 17 candidate explanatory variables (Table S1) based on a priori knowledge of their potential importance to juvenile salmon survival (Grantham et al., 2012; Hwan et al., 2018; Obedzinski et al., 2018; Woelfle‐Erskine et al., 2017). We chose one to two variables describing the following habitat categories: physical dimensions, streamflow, water quality, precipitation, landscape, and fish density. Within categories, we avoided variable pairs that had a Pearson correlation criterion of >0.6 (Figure S2), following Dormann et al. (2013). Our selection resulted in a total of eight explanatory variables that were then standardized by subtracting the variable mean from each value (i.e., centering) and dividing by the standard deviation.

To evaluate the effects of explanatory variables on juvenile salmon survival, we used a generalized linear mixed effects modeling framework (GLMMs; Bolker et al., 2009; Zuur, Ieno, Walker, Saveliev, & Smith, 2009) with binomial distribution and logit link functions using *lme4* package (Bates, Maechler, Bolker, & Walker, 2015) in R (version 3.5.1, R Core Team, 2018). We first created a full model using all eight explanatory variables as fixed effects along with random effects for study reach (*n* = 8 reaches) and year (*n* = 7 years) to account for unobserved variation and repeated measures among reaches and years. Interaction terms of fixed effects were not included to minimize model complexity, and survival estimates with missing measures of explanatory values, such as those from pools that were not stocked with juvenile salmon due to anticipated stream drying, were removed from the analysis. To avoid biased parameter estimates and standard errors created by overdispersion, or variance in observed data that is greater than predicted by the model, we added an observation‐level random effect to each observation of cumulative survival (*n* = 284 observations) to absorb extra‐binomial variation in the data (Harrison, 2015). We determined the effect of each explanatory variable on survival estimates by creating a nested model without that variable using a backward‐stepwise regression procedure (Zuur et al., 2009). Each nested model was compared to the full model using chi‐square tests of model residual deviances. During this process, we removed non‐significant variables (*p* > .05) from the full model and continued testing individual variable effects by comparing nested models and full models until all non‐significant variables were removed (Zuur et al., 2009). We verified the underlying model assumptions and assessed multicollinearity of explanatory variables using the variable inflation factor (vif) function in the R package *car* (Fox & Weisberg, 2011). We extracted estimated coefficients and calculated 95% confidence intervals using Wald's method from GLMMs as measure of variable effect size. We assessed model spatial and temporal transferability using a non‐random, k‐fold cross‐validation procedure (Wenger & Olden, 2012; see Appendix S4 for more details).

## 3 RESULTS

### 3.1 Environmental conditions during study period (2011–2017)

**FIGURE 2:**
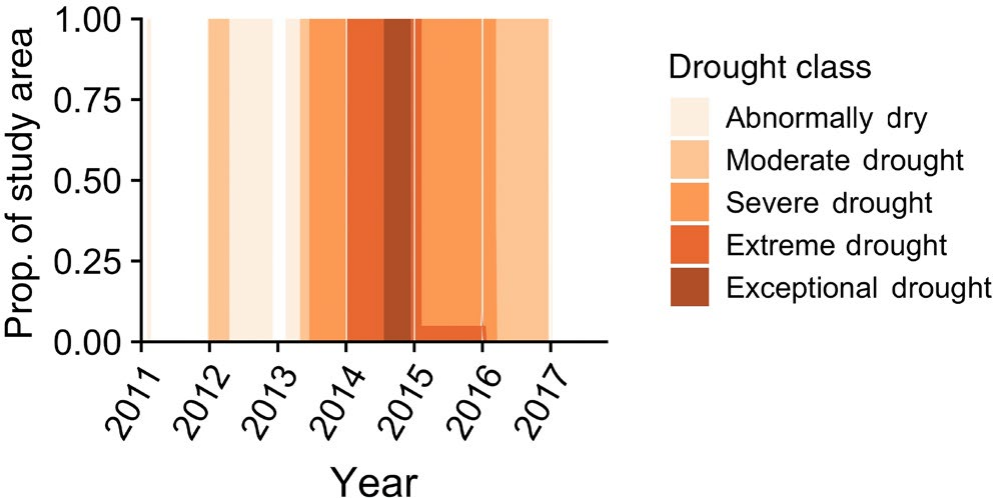
Drought classification between 2011 and 2017 within the Russian River catchment according to the US Drought Monitor (https://droughtmonitor.unl.edu/)

Between 2011 and 2017, the Russian River catchment experienced moderate drought conditions beginning in 2012 (Figure 2), followed by severe drought conditions in 2013 and, by 2014, 100% of the study area was in extreme or exceptional drought (Figure 2). Although drought severity lessened in 2015, much of the study region remained under severe or abnormally dry conditions until 2017. Differences among years in antecedent precipitation, days of pool disconnection, mean streamflow, and maximum water temperature reflected drought (2012–2016) and non‐drought conditions (2011 and 2017; Table S3).

### 3.2 Cumulative juvenile Coho salmon survival

**FIGURE 3:**
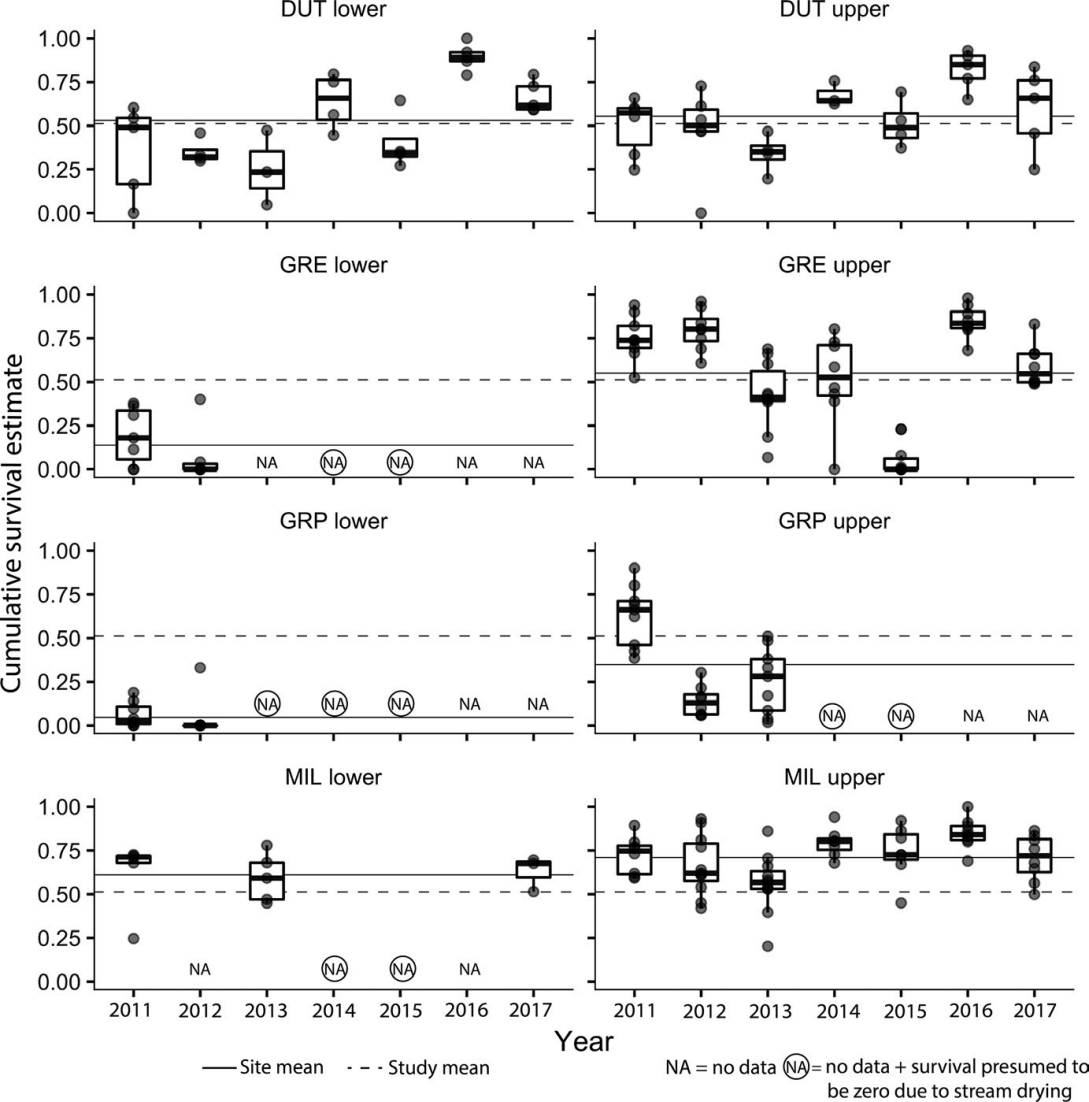
Cumulative survival estimates during 2011–2017 at eight study reaches within the Russian River catchment. Boxplots show median (thick black line), upper and lower quartile, and highest and lowest values within 1.5× the interquartile range. Points represent pool‐level observations within reaches

Mean cumulative survival, averaged across sites and years, during the study was 0.51 ± 0.29 (mean ± *SD* estimated proportion of fish surviving to the end of the dry season; Figure 3). Survival in pools that were stocked with juvenile Coho salmon during the study period was 0.53 ± 0.26 during non‐drought years compared to 0.49 ± 0.32 during drought years. Among study reaches surveyed in all 7 years, MIL upper had the highest mean cumulative survival among study reaches (0.71 ± 0.16) and DUT lower had the lowest mean cumulative survival (0.53 ± 0.26; Figure 3).

**FIGURE 4:**
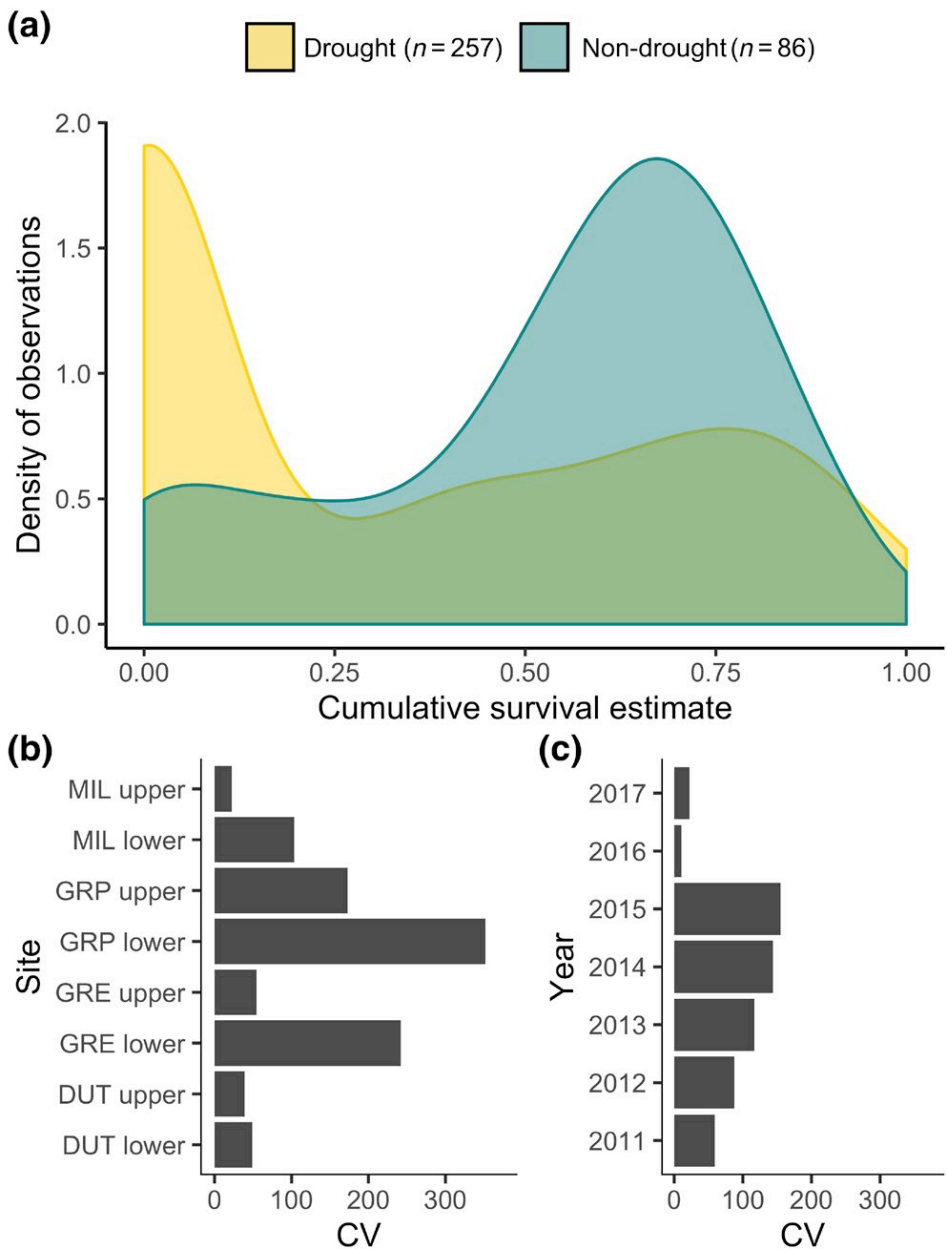
Probability density function illustrating distribution of cumulative salmon survival estimates in stream pools during drought (2012–2016, yellow) and non‐drought (2011 and 2017, blue) years (a). Temporal variability (coefficient of variability within study reaches across years) in cumulative survival estimates at eight study reaches within the Russian River catchment (b). Spatial variability (coefficient of variability across study reaches (sites) during each of the study years) in cumulative survival estimates across study reaches (c). Presumed survival estimates of zero in dry stream reaches were included for visual assessment; however, these estimates are removed from subsequent analysis

The distribution of survival estimates differed significantly between stream pools during drought and non‐drought years (*p* < .001) and was skewed toward zero during drought years (Figure 4a), accounting for pools that were not stocked with salmon due to confirmed stream drying. Temporal and spatial variation (coefficient of variation) in cumulative survival estimates ranged from 22% to 352% across sites and 10%–155% across years, respectively (Figure 4b,c). Mean coefficient of variation was 103% during drought years and 41% during non‐drought years, a 2.5× increase in temporal variability. Nearly half of all pools (47%, 121 of 257 pools) were identified as drought refuges, pools with survival in drought years that was within or above the system‐wide range of survival in non‐drought years. The remaining 53% of pools (136 of 257) were considered ecological traps, with survival estimates below the standard range of system‐wide survival in non‐drought years. In comparison, during non‐drought years, 80% (69 of 86 pools) of pools were designated as “refuges” and 20% (17 of 86 pools) of pools met the criteria to designate them as “ecological traps.”

### 3.3 Effects of explanatory variables on juvenile salmon survival

**FIGURE 5:**
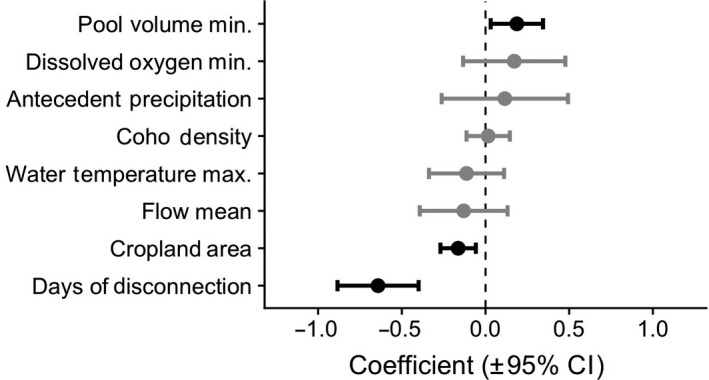
Effect sizes (±95% confidence limits) for eight explanatory variables on cumulative juvenile salmon survival during 2011–2017. Effects sizes estimates are the model coefficients from generalized linear mixed effects models. Black points and confidence bars are statistically significant, whereas grey coloring indicates non‐significant variables (*p* > .05)

Our models indicated that days of disconnection had the greatest influence on over‐summer survival, exhibiting a negative effect on cumulative salmon survival (Table 2; Figure 5). Cropland area had a negative effect, and minimum pool volume had a positive effect on over‐summer survival (Table 2; Figure 5). All other variables were statistically non‐significant, including dissolved oxygen, antecedent precipitation, water temperature, Coho density, and mean daily flow. When transferability of the model was assessed (see Appendix S4 for full results), predicted survival in 50% of study reaches was within 10% of survival estimates, whereas survival in other reaches was, on average, biased by 121%. Predicted survival estimates during drought and non‐drought years had, on average, 100% and 10% bias, respectively.

**TABLE 2:** Results of generalized linear mixed effects models testing the effect of eight explanatory variables on juvenile Coho salmon survival estimates. Bold text indicates variable removed from model has statistical significance on survival estimates

t	Model structure	Variable removed from model	*df*	AIC	Model comparison	ΔAIC	χ^2^	*p* value
1	Coho Density + Dissolved Oxygen Min. + Antecedent Precipitation + Water Temperature Max. + Flow Mean + Pool Volume Min. + Cropland Area + Days of Disconnection (Full Model)	None	12	1,675.6	NA	NA	NA	NA
2	Dissolved Oxygen Min. + Antecedent Precipitation + Water Temperature Max. + Flow Mean + Pool Volume Min. + Cropland Area + Days of Disconnection	Coho Density	11	1,673.7	M2 vs. M1	−1.9	0.059	.809
3	Antecedent Precipitation + Water Temperature Max. + Flow Mean + Pool Volume Min. + Cropland Area + Days of Disconnection	Dissolved Oxygen Min.	10	1,672.8	M3 vs. M2	−0.9	1.146	.284
4	Water Temperature Max. + Flow Mean + Pool Volume Min. + Cropland Area + Days of Disconnection	Antecedent Precipitation	9	1,671.4	M4 vs. M3	−1.4	0.628	.428
5	Flow Mean + Pool Volume Min. + Cropland Area + Days of Disconnection	Water Temperature Max.	8	1,670.5	M5 vs. M6	−0.9	1.070	.301
6	Pool Volume Min. + Cropland Area + Days of Disconnection	Flow Mean	7	1,669.2	M6 vs. M5	−1.3	0.734	.392
**7**	**Cropland Area + Days of Disconnection**	**Pool Volume Min.**	**6**	**1,672.8**	**M7 vs. M6**	**3.6**	**5.505**	**.019**
**8**	**Pool Volume Min. + Days of Disconnection**	**Cropland Area**	**6**	**1,674.2**	**M8 vs. M6**	**1.4**	**6.917**	**.009**
**9**	**Pool Volume Min. + Cropland Area**	**Days of Disconnection**	**6**	**1,705.1**	**M9 vs. M6**	**30.9**	**37.866**	**<.001**

Abbreviation: AIC, Akaike information criterion.

**FIGURE 6:**
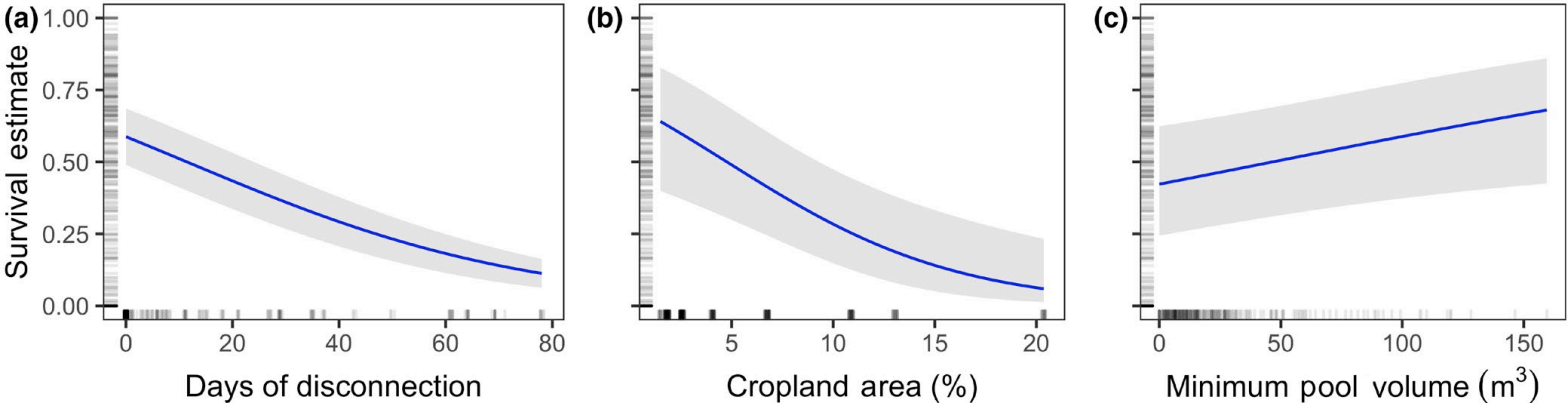
Partial dependence from generalized linear mixed models of juvenile salmon survival. Model estimates (solid lines) and 95% confidence intervals (shading) for days of disconnection (a), cropland area (b), and minimum pool volume (c). Tick marks on the *x*‐ and *y*‐axes indicate values of data observations

Controlling for the effects of all other explanatory variables, an increase from 0 to 78 days of disconnection reduced the cumulative survival rate from 0.59 (0.39–0.70 95% CI) to 0.11 (0.04–0.24; Figure 6a). Estimated cumulative survival was reduced from 0.64 (0.40–0.83) to 0.06 (0.01–0.23) as cropland area increased from 1.5% to 20.0% (Figure 6b). Minimum pool volume during each year had a positive effect on estimated cumulative survival, increasing survival from 0.43 (0.25–0.63) to 0.68 (0.43–0.86) when volume increased from 1.6 to 160 m^3^, respectively (Figure 6c).

## 4 DISCUSSION

In many regions, droughts are predicted to increase in frequency and severity but there remains high uncertainty about potential impacts to species given the uncertain persistence of habitat refuges (Morelli et al., 2017; Thuiller et al., 2004). California's historic multi‐year drought in 2012–2016 provided a unique opportunity to quantify survival during drought and identify key environmental factors affecting survival of endangered juvenile Coho salmon. Drought strongly influenced survival in the study area, with a high proportion of pools (53%) characterized as ecological traps in drought years as compared to non‐drought years (20%). However, the fact that survival in nearly half (47%) of the pools surveyed in drought years was similar to survival during non‐drought periods indicates that habitat within intermittent streams can provide important drought refuges for Coho salmon. Overall, days of disconnection (length of time habitat was fragmented) had the strongest negative effect among a broad suite of potential limiting factors on Coho salmon over‐summer survival.

### 4.1 Key drivers of juvenile salmon survival

Habitat fragmentation is a primary driver of population‐level drought impacts in both terrestrial and freshwater ecosystems (Hwan & Carlson, 2016; Oliver et al., 2015; Perkin, Gido, Costigan, Daniels, & Johnson, 2014). For example, Oliver et al. (2015) found a strong positive association between woodland fragmentation and the sensitivity of the ringlet butterfly (*Aphantopus hyperantus*) to an extreme drought in the UK. In fragmented woodlands, populations were less likely to locate refuges with adequate resources during the drought and thus experienced greater losses and slower recovery (Oliver et al., 2015). Similarly, we found that increased duration of stream pool disconnection negatively influenced pool‐scale juvenile salmon survival. Once disconnected, movement of individuals among pools could no longer occur, preventing salmon from relocating to pools that may have had more suitable environmental conditions as drought conditions worsened over the summer. As suggested by Obedzinski et al. (2018), habitat fragmentation is a “master variable” that encompasses the effects of other variables that potentially limit survival, including water quantity, water quality, food availability, competition, and predation. Although days of disconnection does not provide insight into the proximate cause of salmon mortality in intermittent stream pools, it represents a simple metric for habitat fragmentation that can be measured in the field as a surrogate for predicting survival during extreme drought.

Manipulative experiments could help uncover the proximate mechanisms by which environmental variables influence survival. For example, field‐ or laboratory‐based experiments that mimic drought but allow control over naturally correlated variables, such as temperature and flow, have improved our understanding of population (e.g., Vander Vorste, Malard, & Datry, 2016; Vander Vorste, Mermillod‐Blondin, Hervant, Mons, & Datry, 2017; Walters & Post, 2008) and community responses (e.g., Ledger, Brown, Edwards, Milner, & Woodward, 2013) to river drying. The influence of biotic factors such as competition from other fish (e.g., steelhead trout, Harvey & Nakamoto, 1996) and predation by avian species (Spalding, Peterson, & Quinn, 1995) and mammals (e.g., river otter, Dolloff, 1993) could also be tested using enclosures in a field‐based experimental approach. Moreover, manipulative experiments that allow for testing interactions among biotic and abiotic variables could provide insight into their synergistic or antagonistic effects on populations (Vander Vorste et al., 2017). Interaction between land‐use and habitat fragmentation represents a particularly important variable to test considering that we found a negative association between cropland land‐use and survival, even at <10% cropland area. In our study, we did not quantify interactions among variables owing to sample size limitations; however, we see this as an important next step to identify the drivers of salmon survival.

### 4.2 Variability in survival

High variability in survival within and across study reaches was the most striking results of this study. Variability in survival more than doubled in drought years compared to non‐drought years, highlighting that extreme drought did not uniformly affect habitats across the study area. Some reaches maintained pools with relatively consistent survival estimates (e.g., MIL upper) throughout the study period (CV = 22%), whereas other reaches (e.g., GRP lower) experienced much greater survival variability among pools (CV = 352%; Figure 4). These patterns in survival variability are potentially explained by the high landscape heterogeneity within our study area, which has also been shown to explain variation in species persistence in other ecosystem types (Godfree et al., 2011; Schwantes et al., 2018). Heterogeneity in physical catchment characteristics is a defining feature of Mediterranean climate regions (Cid et al., 2017). Indeed, adjacent catchments within our broader study region have remarkably distinct hydrologic characteristics owing to differences in lithology that influence subsurface water storage (Dralle et al., 2018) and over‐summer streamflow conditions (Larsen & Woelfle‐Erskine, 2018). However, there are currently no regional data available that describe variation in catchment lithology in relation to catchment hydraulics (water storage and runoff properties) and field methods to characterize local substrate and subsurface hydrologic properties within streams remain time‐ and resource‐intensive. As the understanding of catchment and hydrology advances, we expect that the ability to predict summer low‐flow conditions, and therefore salmon survival, will also improve. In the meantime, however, the limited transferability of our model in predicting survival at individual study reaches or years emphasizes that assessing drought impacts on regional populations of sensitive species will remain a challenging task (Moritz & Agudo, 2013; Thuiller et al., 2004).

### 4.3 Drought refuges and ecological traps

Until recently, intermittent streams have been neglected as ecologically important components of freshwater ecosystems (Datry et al., 2018; Marshall et al., 2018); however, our results highlight their importance as drought refuges for an endangered species. Despite unprecedented drought conditions, we estimated that nearly half of the pools were refuges during drought years because they had similar survival to pools assessed in non‐drought years. This result, along with previous findings of increased growth and survival of juvenile Coho salmon in intermittent compared to perennial streams (Wigington et al., 2006), suggests that intermittent stream habitats are vital to Coho salmon persistence along the Pacific Coast. Several other fish species use intermittent streams for breeding (e.g., steelhead and rainbow trout [*O. mykiss*], Erman & Hawthorne, 1976; Hwan et al., 2018, flannelmouth sucker [*Catostomus latipinnis*], and bluehead sucker [*C. discobolus*], Hooley‐Underwood, Stevens, Salinas, & Thompson, 2019) and rearing (e.g., rainbow trout, Erman & Leidy, 1975; Arkansas darter [*Etheostoma cragini*], Labbe & Fausch, 2000), perhaps because they encounter less competition and/or predation compared to perennial streams. Our results emphasize the value of these unique habitats as drought refuges but also underscore their vulnerability to future global change.

For freshwater rivers, increases in flow intermittency and drying associated with extreme drought, in combination with human water demand, likely play an important role in transforming once‐suitable pool habitats into ecological traps. Within our studies sites, we observed anecdotally that pools with alluvial substrate were more likely to fragment and dry and had lower survival compared to pools underlain by bedrock or clay, similar to findings of May and Lee (2004) in Oregon streams. Stream habitats with alluvial substrate are commonly selected for spawning because they contain gravel; however, these habitats are particularly sensitive to water withdrawals from diversions and groundwater pumping, increasing the risk of dewatering redds and stranding juvenile fish (Reiser & White, 1983). Adult salmon preferentially spawn in alluvial substrates where their offspring are more prone high water temperatures and reduced low flows resulting from agricultural water withdrawals (Jeffres & Moyle, 2012). Therefore, environmental cues that influence habitat selection by stream‐rearing fish may be directing them into ecological traps if water withdrawals from direct diversions or streamside wells occur rapidly enough that fish do not have the opportunity to relocate.

### 4.4 Management implications

A continued rise in the number of extreme weather events will no doubt intensify threats to biodiversity (IPCC, 2012) and predicting and mitigating biodiversity loss remains a central challenge to species conservation efforts (Morelli et al., 2017; Thuiller et al., 2004). Our results demonstrate that accurately predicting drought impacts on a species will require going beyond regional‐scale climatological assessments of drought severity. In our case, the Russian River catchment experienced extreme to exceptional drought conditions between 2014 and 2015; however, salmon survival was not uniformly affected by drought and local environmental variables were more influential than watershed‐scale climate and physical features. The type of long‐term, intensive survival studies performed here are expensive and difficult to implement; thus, indicators or proxy variables are needed for assessing drought and climate‐change impacts on sensitive species. Wet‐dry mapping has shown to be a particularly cost‐efficient and effective method for assessing spatial patterns of stream habitat fragmentation (Hwan & Carlson, 2016) and readily lends itself to citizen‐science data collection efforts (Turner & Richter, 2011). As the spatial and temporal extent of such data grows, wet‐dry mapping also holds promise for gaining new insights into where drought refuges and ecological traps occur on the landscape, and the underlying physical mechanisms that influence their persistence. Developing predictive relationships between widely available metrics (e.g., climatic, geological, land‐use) and the occurrence of habitat fragmentation in space and time could then prove a low‐cost means of identifying areas of impairment at a much larger scale, allowing resource managers to better focus limited resources for species recovery.

## Supporting information

Supplementary Material

## Data Availability

R code and data from this study are available upon request to M.O. (mobedzinski@ucsd.edu).
