# Supplementary material for: Refuges and ecological traps: Extreme drought threatens persistence of an endangered fish in intermittent streams
Source: Glob Chang Biol. 2020 May 18;26(7):3834–45. doi: 10.1111/gcb.15116 (PMC7384119; doi:10.1111/gcb.15116)
Supplement: Supplementary file 1 — Supplementary Material [file GCB-26-3834-s001.docx]

**From refuges to ecological traps: extreme drought threatens persistence of an endangered fish in intermittent streams**

Ross Vander Vorste^1^, Mariska Obedzinski^2^, Sarah Nossaman-Pierce^2^, Stephanie M. Carlson^1^, and Theodore E. Grantham^1^

^1^Department of Environmental Science, Policy, & Management, University of California Berkeley, Berkeley, CA USA

^2^California Sea Grant, 600 American Way, Windsor, CA USA

Corresponding author: Ross Vander Vorste, 510-643-4554, [vandervorste.ross@gmail.com](mailto:vandervorste.ross@gmail.com)

**Table S1. Summary of all explanatory variables considered in data analysis**

| **Category** | **Variable name** | **Units** | **Definition (scale)** | N | Mean | SD | Min | Q1 | Median | Q3 | Max | |
| --- | --- | --- | --- | --- | --- | --- | --- | --- | --- | --- | --- | --- |
| hydrology | Days of Disconnection | days | number of days with mean flow < 0.28 L/S (0.1 ft^3^/s) during period of survival estimate (reach) | 284 | 15.3 | 22.9 | 0 | 0 | 2.5 | 27 | 78 | |
| water quality | Maximum Weekly Average Temperature | °C | maximum weekly average water temperature recorded within a reach during period of survival estimate (reach) | 284 | 17 | 0.9 | 15.6 | 16.4 | 16.9 | 17.5 | 18.8 | |
| water quality | Maximum Weekly Maximum Temperature | °C | maximum weekly maximum water temperature recorded within a reach during period of survival estimate (reach) | 284 | 17.9 | 1.4 | 14.7 | 17.1 | 17.8 | 18.8 | 21.6 | |
| water quality | Water Temperature Minimum | °C | minimum water temperature during study period (reach) | 284 | 12.1 | 1.1 | 9.7 | 11.2 | 12.2 | 13 | 14.8 | |
| water quality | Water Temperature Maximum | °C | maximum water temperature during study period (reach) | 284 | 18.7 | 1.3 | 16.5 | 17.8 | 18.7 | 19.6 | 22.5 | |
| water quality | Water Temperature Mean | °C | mean water temperature during study period (reach) | 284 | 15.6 | 0.8 | 14.1 | 15 | 15.5 | 16.1 | 17.8 | |
| water quality | Pool Water Temperature Maximum | °C | maximum water temperature during study period (habitat) | 284 | 15.6 | 1.1 | 12.2 | 15.2 | 15.8 | 16.1 | 18.8 | |
| water quality | Pool Water Temperature Mean | °C | mean water temperature during study period (habitat) | 284 | 14.7 | 0.9 | 12.2 | 14.1 | 14.7 | 15.4 | 16.8 | |
| water quality | Dissolved Oxygen | mg/L | mean spot measurement of dissolved oxygen (habitat) | 284 | 6.6 | 2.2 | 1.1 | 5.1 | 6.9 | 8.6 | 9.8 | |
| water quality | Dissolved Oxygen | mg/L | maximum spot measurement of dissolved oxygen (habitat) | 284 | 6 | 2.6 | 0.2 | 4.3 | 6.4 | 8.3 | 9.8 | |
| physical | Volume Minimum | m^3^ | minimum wetted volume of habitat unit (habitat) | 284 | 24.1 | 27.4 | 0 | 6.8 | 14.8 | 28.1 | 159.5 | |
| physical | Volume Maximum | m^3^ | maximum wetted volume of habitat unit (habitat) | 284 | 29.4 | 29.9 | 0.2 | 10.4 | 18.6 | 34.4 | 173.9 | |
| physical | Water Depth Maximum | cm | maximum water depth of habitat unit (habitat) | 284 | 74.1 | 26.5 | 10.4 | 54.9 | 72.7 | 93 | 179.8 | |
| physical | Water Depth Minimum | cm | minimum value of maximum water depth of habitat unit (habitat) | 284 | 67.6 | 28.3 | 0 | 48.2 | 67.1 | 87.9 | 179.8 | |
| landscape | Catchment Area | km^2^ | catchment drainage area (reach) | 284 | 14.3 | 9.4 | 7.4 | 8 | 10.4 | 14.9 | 43 | |
| landscape | Slope | % | slope of study reach (reach) | 284 | 0.9 | 0.5 | 0.3 | 0.4 | 0.7 | 1.1 | 1.9 |  |
| landscape | Cropland Area | % | percent area of catchment with croplands landuse (reach) | 284 | 5.6 | 5 | 1.5 | 1.8 | 2.6 | 6.7 | 20.4 | |
| landscape | Number of Buildings | — | number of structures detected within the catchment (reach) | 284 | 819 | 1910.9 | 97 | 122 | 208 | 931 | 8760 | |
| climate | Antecedent Precipitation | mm | cumulative precipitation for the months of Oct. – May prior to the period of survival estimate (reach) | 284 | 1063.0 | 337.9 | 714.8 | 829.0 | 1032.1 | 1178.1 | 2267.2 | |
| climate | Recent Precipitation | mm | cumulative precipitation during period of survival estimate (reach) | 284 | 63.5 | 69.5 | 0 | 17.3 | 33.1 | 105.7 | 307.2 | |
| biology | Coho Density | ind./m^3^ | Estimated density of juvenile coho salmon within habitat unit (habitat) | 284 | 2 | 2.3 | 0 | 0.8 | 1.4 | 2.4 | 30 | |
| hydrology | Flow Mean* | m^3^ s^-1^ | mean daily water discharge measured within a reach during period of survival estimate (reach) | 284 | 33 | 33.7 | 0.3 | 9.4 | 21.3 | 44 | 127.1 | |
| hydrology | Flow Minimum* | m^3^ s^-1^ | minimum flow recorded during period of survival estimate (reach) | 284 | 4.3 | 6.5 | 0.1 | 0.2 | 0.3 | 6.3 | 24.8 | |
| hydrology | Flow Maximum* | m^3^ s^-1^ | mean of daily maximum water discharge within a reach during period of survival estimate (reach) | 284 | 660 | 2010.3 | 0.3 | 60 | 117.8 | 297.9 | 11315.7 | |

**Table S2: Pearson correlation matrix of physical, hydrologic, water quality, precipitation, and landscape variables considered in data analysis.**

| **Pearson correlation among physical habitat variables** | | | | | |  |  |  |  |
| --- | --- | --- | --- | --- | --- | --- | --- | --- | --- |
|  | MinVolume_pool | MaxVolume_pool | MaxDepth_pool | minMaxDepth_pool | | | |  |  |
| MinVolume_pool | 1.0 | 0.9 | 0.5 | 0.6 |  |  |  |  |  |
| MaxVolume_pool | 0.9 | 1.0 | 0.5 | 0.5 |  |  |  |  |  |
| MaxDepth_pool | 0.5 | 0.5 | 1.0 | 0.9 |  |  |  |  |  |
| minMaxDepth_pool | 0.6 | 0.5 | 0.9 | 1.0 |  |  |  |  |  |
| **Pearson correlation among hydrologic habitat variables** | | | | | | |  |  |  |
|  | Ndays_disconnect | cMeanFlow | cMinFlow | cMaxFlow | |  |  |  |  |
| Ndays_disconnect | 1.0 | -0.5 | -0.4 | -0.2 |  |  |  |  |  |
| cMeanFlow | -0.5 | 1.0 | 0.5 | 0.4 |  |  |  |  |  |
| cMinFlow | -0.4 | 0.5 | 1.0 | 0.0 |  |  |  |  |  |
| cMaxFlow | -0.2 | 0.4 | 0.0 | 1.0 |  |  |  |  |  |
| **Pearson correlation among water quality variables** | | | | | |  |  |  |  |
|  | MWAT | MWMT | MinTemp | MaxTemp | MeanTemp | MaxTemp_pool | MeanTemp_pool | MeanDO_pool | MinDO_pool |
| MWAT | 1.0 | 0.9 | 0.0 | 0.9 | 0.8 | 0.3 | 0.4 | 0.3 | 0.2 |
| MWMT | 0.9 | 1.0 | -0.2 | 0.9 | 0.6 | 0.2 | 0.3 | 0.3 | 0.2 |
| MinTemp | 0.0 | -0.2 | 1.0 | -0.2 | 0.3 | 0.2 | 0.5 | -0.2 | -0.2 |
| MaxTemp | 0.9 | 0.9 | -0.2 | 1.0 | 0.7 | 0.3 | 0.3 | 0.3 | 0.2 |
| MeanTemp | 0.8 | 0.6 | 0.3 | 0.7 | 1.0 | 0.6 | 0.7 | 0.0 | 0.0 |
| MaxTemp_pool | 0.3 | 0.2 | 0.2 | 0.3 | 0.6 | 1.0 | 0.8 | -0.3 | -0.3 |
| MeanTemp_pool | 0.4 | 0.3 | 0.5 | 0.3 | 0.7 | 0.8 | 1.0 | -0.3 | -0.3 |
| MeanDO_pool | 0.3 | 0.3 | -0.2 | 0.3 | 0.0 | -0.3 | -0.3 | 1.0 | 1.0 |
| MinDO_pool | 0.2 | 0.2 | -0.2 | 0.2 | 0.0 | -0.3 | -0.3 | 1.0 | 1.0 |
| **Pearson correlation among precipitation variables** | | | | | |  |  |  |  |
|  | Precip_antecedent | Precip_sum | |  |  |  |  |  |  |
| Precip_antecedent | 1.0 | 0.1 |  |  |  |  |  |  |  |
| Precip_sum | 0.1 | 1.0 |  |  |  |  |  |  |  |
| **Pearson correlation among landscape variables** | | | | |  |  |  |  |  |
|  | CatchArea | PctSlope | PctCropland | NBuildings | | |  |  |  |
| CatchArea | 1.0 | -0.3 | 0.4 | 0.8 |  |  |  |  |  |
| PctSlope | -0.3 | 1.0 | 0.3 | -0.2 |  |  |  |  |  |
| PctCropland | 0.4 | 0.3 | 1.0 | 0.7 |  |  |  |  |  |
| NBuildings | 0.8 | -0.2 | 0.7 | 1.0 |  |  |  |  |  |

**Table S3. Mean** ± **standard deviation (s.d.) of variables used in models to explain juvenile salmon survival.**

|  | 2011 | | 2012 | | 2013 | | 2014 | | 2015 | | 2016 | | 2017 | |
| --- | --- | --- | --- | --- | --- | --- | --- | --- | --- | --- | --- | --- | --- | --- |
| Variable name (units) | mean | s.d. | mean | s.d. | mean | s.d. | mean | s.d. | mean | s.d. | mean | s.d. | mean | s.d. |
| Antecedent Precipitation (mm)* | 1,154.1 | 58.2 | 786.7 | 47.9 | 948.4 | 56.2 | 760.2 | 33.5 | 912.3 | 100.0 | 1,246.5 | 146.2 | 1,849.9 | 297.8 |
| Coho Density (individuals m^3^) | 2.6 | 3.9 | 1.7 | 1.6 | 1.1 | 0.7 | 2.6 | 2.3 | 2.3 | 1.8 | 1.8 | 1.1 | 1.7 | 1.1 |
| Cropland Area (%) | 8.0 | 6.2 | 8.4 | 6.2 | 4.8 | 3.7 | 3.3 | 1.8 | 3.3 | 1.8 | 3.4 | 1.8 | 3.2 | 1.8 |
| Days of Disconnection (days)* | 5.8 | 11.2 | 12.8 | 16.6 | 30.3 | 29.2 | 29.1 | 24.9 | 33.7 | 28.9 | 0.0 | 0.0 | 0.3 | 0.5 |
| Dissolved Oxygen Min. (mg L^-1^) | 6.8 | 1.7 | 5.9 | 2.9 | 5.3 | 3.0 | 4.7 | 2.4 | 4.7 | 2.9 | 7.3 | 1.5 | 6.5 | 2.3 |
| Pool Volume Min. (m^3^) | 24.1 | 25.5 | 16.1 | 20.0 | 28.1 | 32.4 | 22.1 | 27.8 | 17.7 | 22.1 | 28.2 | 25.9 | 38.3 | 35.6 |
| Streamflow Mean (m^3^ day)* | 68.3 | 33.0 | 19.7 | 11.7 | 18.3 | 21.8 | 6.5 | 2.8 | 7.7 | 4.5 | 23.9 | 6.2 | 63.0 | 42.7 |
| Water Temperature Max. (°C)* | 15.7 | 0.6 | 14.9 | 1.4 | 15.8 | 0.3 | 15.8 | 0.3 | 16.2 | 0.5 | 14.2 | 0.7 | 16.8 | 0.9 |

Asterisk indicates variable mean is different between drought (2012–2016) and non-drought (2011 & 2017) years (Welch’s t-test, p < 0.05).

**Appendix S4: Description of methods and results for the assessment of model spatial and temporal transferability.**

*Assessing model spatial and temporal transferability*

We followed a non-random, k-fold cross-validation procedure to evaluate model transferability to different stream reaches and years (Wenger and Olden 2012). To evaluate spatial transferability, we separated our observations based on reach location and used all but one of these reaches to train our final model after removing reach as a random effect. We then use the trained model to make predictions of survival for the remaining reach and compared predictions to the observed survival estimate. To evaluate temporal transferability, we repeated this same procedure using year of observation to separate data into training and test datasets. For both procedures, we report the mean bias, positive or negative, of our predicted survival estimates to observed survival estimates as an indication of model transferability.

When separating observations of cumulative survival by study reach, the most parsimonious model of cumulative salmon survival estimated survival with less than 10% bias, based on predicted-observed estimates, in half of the study reaches. Estimate bias was higher in DUT lower, GRE lower, GRE upper, and GRP upper (bias mean = 121%, min = 34%, max = 270%).

When separating observations of cumulative survival by study year, the most parsimonious model of cumulative salmon survival estimated survival with less than 10% bias, based on predicted-observed estimates, in two of the study years (2011 and 2017). Estimate bias was higher in 2012–2016 (bias mean = 100%, min = 23%, max = 349%).

References for Appendix S4

Wenger S. J., Olden J. D. (2012) Assessing transferability of ecological models: an underappreciated aspect of statistical validation. *Methods in Ecology and Evolution,* 3, 260-267.
